# A critical assessment of the WHO responsiveness tool: lessons from voluntary HIV testing and counselling services in Kenya

**DOI:** 10.1186/1472-6963-9-243

**Published:** 2009-12-22

**Authors:** Mercy K Njeru, Astrid Blystad, Isaac K Nyamongo, Knut Fylkesnes

**Affiliations:** 1 Centre for Public Health research, Kenya Medical Research Institute, Nairobi, Kenya; 2 Centre for International Health, Faculty of Medicine and Dentistry, University of Bergen, Bergen, Norway; 3 Institute of Anthropology, Gender and African studies, University of Nairobi, Nairobi, Kenya; 4 Department of Public Health and Primary Health Care, Faculty of Medicine and Dentistry, University of Bergen, Bergen, Norway

## Abstract

**Background:**

Health, fair financing and responsiveness to the user's needs and expectations are seen as the essential objectives of health systems. Efforts have been made to conceptualise and measure responsiveness as a basis for evaluating the non-health aspects of health systems performance. This study assesses the applicability of the responsiveness tool developed by WHO when applied in the context of voluntary HIV counselling and testing services (VCT) at a district level in Kenya.

**Methods:**

A mixed method study was conducted employing a combination of quantitative and qualitative research methods concurrently. The questionnaire proposed by WHO was administered to 328 VCT users and 36 VCT counsellors (health providers). In addition to the questionnaire, qualitative interviews were carried out among a total of 300 participants. Observational field notes were also written.

**Results:**

A majority of the health providers and users indicated that the responsiveness elements were very important, e.g. confidentiality and autonomy were regarded by most users and health providers as very important and were also reported as being highly observed in the VCT room. However, the qualitative findings revealed other important aspects related to confidentiality, autonomy and other responsiveness elements that were not captured by the WHO tool. Striking examples were inappropriate location of the VCT centre, limited information provided, language problems, and concern about the quality of counselling.

**Conclusion:**

The results indicate that the WHO developed responsiveness elements are relevant and important in measuring the performance of voluntary HIV counselling and testing. However, the tool needs substantial revision in order to capture other important dimensions or perspectives. The findings also confirm the importance of careful assessment and recognition of locally specific aspects when conducting comparative studies on responsiveness of HIV testing services.

## Background

The World Health Organization (WHO) advises that evaluation of performance of any health action should be centred on the 3 fundamental goals of a health system: improving health, enhancing responsiveness to the user's expectations, and assuring fairness of any financial contribution [1-4]. In this context, patient surveys aimed at generating knowledge to make health services more responsive to the user's needs and expectations are becoming increasingly important [5]. Responsiveness in the context of health systems has been defined as "the outcome that can be achieved when health institutions and institutional relationships are designed in such a way that they are cognisant of and respond appropriately to the universally legitimate expectations of individual" [6,7]. This very broad definition can be viewed from 2 perspectives. Firstly, the user of the health-care system is seen as a consumer where greater responsiveness becomes a means of attracting consumers. Secondly, responsiveness is seen safeguarding the rights of patients to adequate and timely care [6].

A responsive health system needs to contribute to the enhancement of health by creating a conducive environment that increases the likelihood of individuals seeking care earlier, increases the openness in their interactions with the health-care providers, and improves their assimilation of health information [7]. Responsive health systems can contribute by reducing barriers to the use of health services, making responsiveness a strong determinant of trust in them [8]. Two major components have been defined by WHO in attempts to measure responsiveness, namely respect for persons, which captures aspects of individual interaction with the health system, and client orientation, which includes several aspects of consumer satisfaction [1,3,6]. WHO also developed 7 elements as the central elements needed to measure the responsiveness of a health system and consequently validated a questionnaire that was used to measure levels of responsiveness in surveys [3,9]. This tool has since been employed in several studies [10-12]. Responsiveness is one of the central parameters in health-care performance [4], making surveys measuring responsiveness instrumental in providing evidence that can guide resource allocation and management strategies [6].

The comparability of different health systems using a single tool to measure performance has been questioned [13]. Studies conducted on health-related responsiveness in Turkey and Taiwan for example, found that recognition of the value of culturally specific aspects, demographic structures and country specific factors should be taken into account when assessing responsiveness. Their advice is that responsiveness ranking countries should be done on the basis of tools that take into account the views of their own citizens [10,12].

The responsiveness of health systems is of particular importance in the context of HIV, due to the heavy stigma associated with this infection. In these contexts, highly responsive health systems are of vital importance for trust and acceptability. A case of concern is the low uptake of voluntary HIV counselling and testing (VCT). VCT has been defined as a confidential process by which people undergo individual counselling to enable them to make an informed choice about being tested voluntarily for HIV [14], and to consider their own HIV related risk. These services are pivotal in meeting the commitment of "universal access to prevention, treatment and care" in an HIV context [14-16]. Despite the importance given to VCT, studies from sub-Saharan Africa have shown that while readiness for VCT is high, utilization is still low, even in places where services are readily available [17,18]. Uptake is particularly low when offered from centres located in general health clinics. Limited trust has been suggested as part of the cause of poor acceptability (the difference between intention and actual use) of VCT [17,19-22]. In a context of stigma and high sensitivity, responsiveness emerges as a highly relevant concept in evaluating HIV prevention and care programs.

About 8% of Kenyan adults (15-49 years) are estimated to have HIV [23]. A wide range of preventive, care, support and treatment interventions have been instituted over the past 20 years to meet the epidemic. Among them is relatively rapid scaling up in HIV counselling and testing services [24]. However, little is known about how these VCT services respond to the expectations and needs of the people. In this study we investigated the applicability and relevance of the WHO developed elements (dignity, autonomy, confidentiality, prompt attention, quality of basic amenities and choice of providers) proposed to measure responsiveness, within the context of VCT services as a district level in Kenya.

## Methods

### Study design

The study was initially intended to use quantitative methods, but the pilot study indicated the need for mixed methods so as to explore other elements of responsiveness that were not captured by the closed questions within the quantitative survey. Mixing quantitative and qualitative methods can be used to add insights likely to be missed when only a single method is used and to increase generalizability to the results [25]. A concurrent nested study design [26] was adapted to enable the researchers to gain broader perspectives of responsiveness, by adding a qualitative open-ended question to the quantitative questionnaire. This type of design enables the collection of quantitative and qualitative data during one phase and it involves interviewing the same persons using different techniques, which in turn could help to identify measurement and methodological problems [26,27].

The study was conducted from October to November 2007 in Malindi district, in Kenya where the EU-funded five year intervention study "REsponse to ACcountable priority setting for Trust in health systems" (REACT) is being conducted [28]. The intervention being applied is an explicit ethical framework for legitimate and fair priority setting, accountability for reasonableness (AFR). The values being focused in the evaluation of the intervention are quality, equity and trust [28]. In addition to Kenya, the REACT research project is ongoing in Tanzania and Zambia. The present paper is a result of a study within the frame of REACT. Malindi district was chosen due to the relative similarity to the other two districts within the REACT project in terms of disease burden, health system and population [28]. HIV control programmes were defined as one of the several evaluation domains of the project due to the high HIV prevalence in the project countries. The adult (15-49) HIV prevalence is between 15%-17% in Malindi [29].

### Research tools

#### The quantitative study

The World health organization developed and validated a questionnaire to measure responsiveness that incorporates the 7 elements indicated in Table 1, with varying number of questions related to each element [30]. The present study applied the tool that had been implemented in a previous study (among key informants in 35 countries) [3], but tailored it to address 6 of the 7 elements to fit the study setting. The element '*access to social support networks during care' *was omitted because the questions within this section were not deemed applicable for VCT. These questions were relevant in the context of inpatient care [4,7].

**Table 1:** Elements as defined in the WHO responsiveness concept [4,7]

*Element*	Question Handles (Sub-elements)
Dignity	The element implies that individuals are treated with respect by being welcomed at the health-care unit and addressed respectfully. It also implies being treated with concern, and being examined in a manner that respects the client's privacy and the right of individuals with infectious diseases such as HIV to be safeguarded.

Autonomy	This element deals with involvement in decision making, and assumes that this can only happen if the users are provided with relevant information, consulted on preferences, and that patients' consent is sought before any proceeding. It also implies that respect is observed on the right of patients of sound mind to refuse treatment.

Confidentiality	This element of responsiveness is related to high maintenance of confidentiality of any information that is provided by the patient, confidentiality of medical records and information about individuals, and privacy during consultations by health providers.

Prompt Attention	This element is defined as care provided readily and as soon as necessary. It includes short waiting-times for treatment or consultations, short-lists for consultations, reasonable waiting-times for appointments, fast care for emergencies as well as the accessibility of the health facility.

Quality of Basic Amenities	This element deals with the extent to which the health facility's physical infrastructure is welcoming and pleasant. It mainly includes clean surroundings, maintenance, adequate furniture, sufficient ventilation, clean water, clean toilets and clean linen.

Choice of Provider	This element is related to the health-care institutions and health providers. It is defined as the power or opportunity to the selection of a provider which requires more than one option. It deals with patients being able to access health services without much difficulty, ability to choose a health-care provider within a health-care unit, individuals being able to get a second opinion, and ability of individuals to get appropriate specialist care.

Social Support	In Hospitals: visits, having special foods, religious practices.

To ensure equivalence of the original version, a bilingual English to Swahili translator with medicine, epidemiology and public health background (who also has an understanding of the local language in Malindi area) was asked to perform a back-translation after the English WHO questionnaire had been translated to Swahili by a professional. Where differences were noted, the issues were discussed among the 2 translators, as well as with Swahili speakers at the study area.

Social demographic questions that were included in the questionnaire for the VCT user's i.e. individuals who have utilized VCT services and for the health-care providers whom we refer to as 'providers', captured information on the type of VCT visited, geographical location, sex and age. Marital status was also mapped in the questionnaire for the VCT users.

The health-care providers' questionnaire rated the sub-elements on a 4 point scale ranging from 'never' (1) to 'always' (4) or 'very poor' (1) to 'very good' (4). To measure the perceived importance of the elements of responsiveness, the study participants (both users and providers), were asked to indicate how important they felt the WHO elements or aspects were on a scale from 0 (not at all important) to 10 (very important). The questionnaire was administered as an exit questionnaire to the users of VCT to generate responses based on their immediate experience with the facilities. A total of 328 VCT users and 36 health-care providers were interviewed.

#### The qualitative study

The qualitative part of the study consisted of an open-ended question that was added to the quantitative questionnaire as well as the use of observations. The open-ended question was added to allow informants to respond in their own words which in turn permit understanding of responsiveness as seen by the informants. The question sought to bring up potential issues of relevance for responsiveness that were not captured by the existing responsiveness tool. Observation was used in this study to add to our informant's responses. The open question was phrased thus, "in your view are there any other characteristics (other that the ones we have discussed) that you think should be included in a responsive VCT?" A number of probe questions were added to generate more in-depth information on this topic. For example we asked: "What should be done to make VCT services more responsive and to increase its utilization? Probe: Why do you think so? Who should be responsible?" Further probes were formulated depending on the initial responses given. The responses from the users were recorded through hand writing. As the informants felt more comfortable when their responses were not electronically recorded, 4 of the 36 provider's responses were tape recorded.

### Study setting

The survey was carried out among all the VCT counsellors or health-care providers who were available as well as among all the users of VCT services in the 15 VCT existing in the district at the time of study (October - November 2007). Most of the VCT facilities in Malindi are integrated or situated within health facilities where there are many other points of provider-initiated HIV testing and counselling such as maternal and child health clinics, tuberculosis clinics and general outpatient services among others. HIV testing and counselling at the VCT facility is mainly client initiated. Anonymity - as documented in the use of code numbers and mother's names was practiced in the Malindi VCT facilities to ensure confidentiality. Those who tested HIV positive were provided with further referral following the ministry of health recommended procedure in order to maintain confidentiality.

### Study population

A total of 331 VCT users were asked to participate, out of which 328 accepted to be interviewed for the quantitative part. All the 36 counsellors approached agreed to participate. The study participants were recruited after being informed about the study focus, the voluntary nature of the study and after assurance of confidentiality and anonymity. The inclusion criteria for the 'providers' was VCT staff; while the users were individuals above 18 years who had utilized existing VCT service. The open ended question was addressed to a total of 264 out of the 328 users. Of the remaining 64 participants, 39 of them declined to be interviewed further because they were satisfied with the existing VCT services and 25 did not wish to respond further.

### Data collection

#### The interviews and Observations

All the interviews were carried out face-to-face by the first author [MKN] with assistance from trained and experienced local interviewers. Data was obtained through exit interviews which were seen as the best option for this study in order to avoid recall and recognition bias around perceptions, attitudes and experiences encountered by the VCT users. The quantitative section of the interviews lasted between 30 minutes and one hour, while the qualitative interviews (consisting of the open ended question), took another 30 minutes or more. Among the health providers, English was the primary language of communication, with Swahili words occasionally employed where necessary, while English, Swahili and some Giriama (local language) words were used among the VCT users. All interviews were conducted in a private area or a room provided within the VCT vicinities.

The collected information was kept in a locked cupboard to ensure confidentiality. In addition to the interviews, individual observational field notes were written on a daily basis. These consisted of:exact locations of the VCT, observed dynamics amongst people using VCT, reception of clients by providers, gestures by users, the VCT infrastructure, sitting arrangements before a VCT session, type of health provider and information, education and communication (IEC) material available at the site.

### Data Analysis

Quantitative data was analyzed using SPSS version 15 for Microsoft Windows. Graphs and tables were produced using Microsoft Excel and Microsoft Word. In accordance with the WHO approach in a previous study [3], performance outcomes were dichotomised into good performance (combining responses very good and good or always and usually) and poor performance (combining responses poor and very poor or never and sometimes). For the user's data set, 'yes' responses were classified as good performance and 'no' as poor. The importance question was grouped into very important (combining responses 9 and 10), important (5-8) and less important (1-4).

The open-ended responses were mainly translated to English and thematic analysis was employed. Thematic analysis has the following stages that were adapted in the analysis; familiarization with the material, identification of a thematic framework, indexing or coding, mapping and interpretation [31]. The coding process was conducted so as to identify specific pieces of data which were relevant to the responsiveness elements in order to add information.

### Ethical aspects

Scientific and ethical approval was obtained from the Kenya Medical Research Institute (KEMRI) and the National Ethical Review Committee (NERC) of Kenya prior to conducting the study (KEMRI SSC number 1273). Written informed consent was obtained from all participants prior to the interviews.

## Results

### Demographic characteristics

Forty-four percent of the providers were based at the rural VCT centres, with 14% in peri-urban and 42% in urban centres. Among the users, 31% attended the rural, 18% the peri-urban and 51% the urban VCT centres. There were more female users (65%) than male (35%) as indicated in Table 2.

**Table 2:** Demographic characteristics of the respondents

*Characteristic*	*Providers*		*Users*	
	
	n	%	n	%
Setting				
Rural	16	44.4	100	31
Peri-urban	5	13.9	60	18
Urban	15	41.7	168	51

Sex				
Male	20	55.6	115	35
Female	16	44.4	213	65

Age				
18-24	3	8.3	88	27
25-29	11	30.6	68	21
30-34	4	11.1	44	13
35-39	9	25.0	44	13
40 and more	9	25.0	66	20

Type of VCT				
Integrated^1^	32	88.9	268	82
Stand alone^2^	4	11.1	17	5
Mobile clinic^3^	-	-	43	13

Marital status				
Married	-	-	178	55
Single never married	-	-	79	22
Cohabiting/living as married	-	-	38	10
Separated/divorced	-	-	24	7
Widowed	-	-	20	6

^1^Integrated: VCT facilities that are located within a health facility or hospital, ^2^stand alone: a VCT that is not within a facility. ^3^Mobile seek users in the community on certain days.

The structuring of the findings is done by the responsiveness elements which have further been defined in Table 1. The quantitative and the qualitative findings pertaining to the same element are presented within the same section. Responses from the open-ended questions are referred through direct quotes from study informants. Findings based on the observation or field notes are referred to separately.

**Figure 1:**
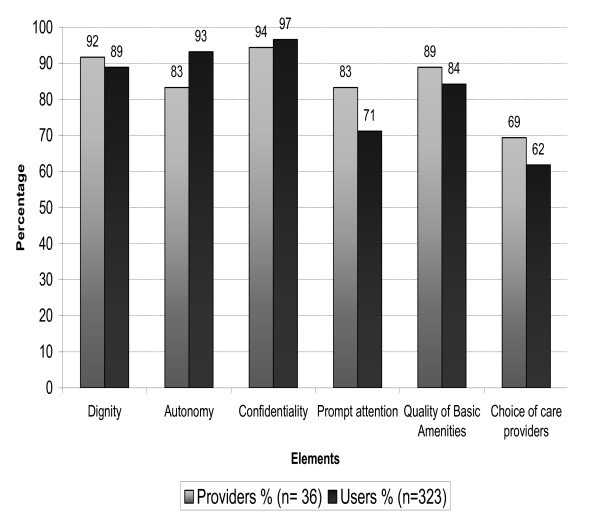
**Proportions of Study participants who felt that responsiveness elements were very important^1^**. ^1^Very important: cut-off point in the rating from 1-10 was 9 or above.

***Confidentiality ***was regarded by almost all users (97%) as well as by the providers (94%) as a very important factor to be considered in VCT (Fig. 1). All the providers reported that the confidentiality of information from medical records and consultation sessions was highly observed, while 98% of the users reported that confidentiality during consultations was highly met at the VCT centres (Tables 3)

**Table 3:** Proportions reporting good performance* of the responsiveness sub-elements at VCT

*Element*	*Sub-elements*	*Providers %*	*Users %*
Dignity^1^	Treated with respect	100	99
	Patients encouraged to discuss concerns freely	98	97
	Patients encouraged to ask question about the disease (prevention treatment and care)	97	91
	Patients privacy during testing and counselling	100	95
	Patients privacy during counselling		86
			
Autonomy^1^	Patients provided with information on prevention and care of HIV	100	95
	Patients consent sought before testing	100	99
	Patient Counselled		98
			
Confidentiality^1^	Confidentiality observed during consultations	100	98
	Confidentiality of information observed	100	-
	Confidentiality of medical records	100	-
			
Choice of care^1 ^provider	Choice between health care providers at VCT	50	29
	Choice between VCT	64	64
			
Quality of basic amenities^2^	Cleanliness of VCT	97	96
	Furniture availability	89	81
	Maintenance	95	93
	Access to clean water	94	-
	Cleanliness of toilets	91	-
	Testing Kit	100	-
			
Prompt attention^3^	Waiting times	83	76

*Good performance: cut-off point in dichotomizing ratings on ^1^always and, usually, ^2^very good and good and ^3^30 minutes or less.

Both the open-ended responses and the observations to some extent conflicted with the impression that a very high degree of confidentiality was assured at the VCT centre. The qualitative data revealed issues pertaining to confidentiality and privacy that were not captured by the responsiveness tool. Various aspects of the maintenance of privacy and confidentiality were pointed out as lacking. One aspect that was brought up in a number of responses, was the unease experienced at the possibility of meeting health-care providers known by the user, as expressed in the following statement: "*People are scared of going to the VCT because they are afraid that they will know the counsellor and he/she might tell the results to others" (a 26 year old female urban VCT user)*. Besides such expressions of the importance of anonymity, a substantial desire to maintain privacy during VCT consultations also emerged in a large number of statements pertaining to the physical arrangement of the VCT facility. An informant noted that *"The VCT room should not be located next to the consultation room for confidentiality reasons. It should be situated in a private area where one is not seen by everyone when entering or coming out" (a 38 year female urban VCT user)*. Field observations revealed that at most of the VCT centres, the testing and counselling room was located just opposite or next to the consultation room, as shown in additional file 1. On some occasion, it was possible to hear conversations taking place in the next room. The concept of confidentiality is further illustrated by the following statement from an 18 year male VCT user from a rural setting "*The providers should find a way of making the VCT consultations more private and confidential through more mobile VCT services"*.

***Dignity:*** A large majority of both the users (89%) and the providers (92%) indicated this element to be very important (Fig. 1). Most of the sub-themes within this element (such as being treated with respect, being encouraged to discuss concerns freely, being encouraged to ask questions and observation of privacy during testing and counselling) were reported to be met, ranging between 97% and 100% among the providers and between 91% and 99% of the users (Table 3). Hence, like confidentiality, dignity was reported to be observed to a very high degree at the VCT facilities.

In relation to this element, the informants brought up aspects that were not captured by the responsiveness tool. Some of the informants pointed out that the providers should greet waiting clients as a gesture of respect. The concerns that were raised were particularly related to the lack of making clients feel relaxed outside the VCT room. Informants commented in a number of ways on a strong unease experienced upon arrival at the VCT centres. *"The providers need to respect the clients by showing them courtesy. For example asking kindly what one is coming for upon arrival. Then they can direct the person to the VCT room" (a 26 year urban VCT user)*.

The physical arrangement of the facility also emerged in the assessment of the observance of dignity at the VCT centre. At times, the location of the entrance, the reception area and the VCT rooms made it necessary for the arriving users to move into highly congested sitting or reception areas in order to ask for directions. A couple of times the users asked the research team for assistance in finding the VCT room. If there was no one present at the entrance to assist, it was quite likely that apprehensive users would not move into the crowd and would shy away from the facility. A respectful health-care provider that would greet the user and discretely direct the user to the VCT room upon arrival was commonly missed.

***Autonomy:*** Again, a large majority of the users (93%) reported this element to be very important. A slightly lower proportion (83%) of the providers found autonomy to be very important (Fig. 1). As with the assessment of confidentiality and dignity, almost all of the providers and users reported that autonomy was highly endorsed at the VCT centres (Table 3).

Some important insights were gained from the qualitative responses. A large number of users reported that they received too little information to be able to make informed decisions and they missed more posters and reading materials on HIV. Take-home material in the local language was particularly asked for so that the topics brought up could be dwelt on in quiet and less stressful surroundings. The issue of the use of the local language was also pointed out in a counselling context where informants needed a language that would make complex messages clear and more understandable to the user. "*The counsellors should be able to speak the local languages because they give us a lot of information. As for me, I did understand some things but not all that the counsellor said" (18 year old female user, peri-urban VCT user)*.

***Quality of basic amenities:*** Like the other elements, the majority of the users and providers scored highly in terms of the importance of this element(84% and 89%). Both groups of the respondents gave high scores on the performance of the element, except those on clean water and toilets which users scored relatively low, primarily because they did not know whether such facilities existed (Table 3).

When we asked the WHO-defined questions on basic amenities, questions and expressions indicated that the informants did not see anything particularly wrong with neither hygiene or maintenance nor content of the current VCT centres, which all had the same lay-out. Most of the informants came from humble homes (additional file 2), and the appearance of the VCT structures emerged to be satisfactory. There was general concern about space, however, especially where it was needed to ensure confidentiality. The providers were also concerned about the lack of space: *"Sometimes we have many clients and there is only one room. If we could have extra rooms where we could have four sessions going on at the same time, this would reduce the waiting time so that you don't lose people (a female counsellor, urban VCT)*. The field-notes recorded that space was sometimes so limited that the counsellors had to compromise confidentiality. On one occasion a counsellor had to counsel a client outside the VCT room because another was being attended to inside the room. The person being counselled outside looked uneasy because the mothers queuing at the clinic opposite were all curious about what was being said.

***Prompt Attention:*** A higher proportion of the provider's (83%) indicated this element to be very important than the users (71%). 76% of the users reported that they waited 30 minutes or less before consultation, while 83% of the providers reported that most users waited 30 minutes and less (Table 3).

The open-ended responses however indicated substantial variation in waiting times, and that these naturally were related to the number of providers available and availability of space. A point brought up by many of the informants was the need to have the counselling services extended to later hours to allow people to visit the VCT centre at a convenient time.

" The centre should be operating up to late hours so that those who are shy to be seen can come for testing when they are comfortable" (a 27 year male urban VCT user)

***Choice of Provider:*** In terms of importance, this element was assessed as the least important of the WHO elements. Only 62% of the users and 69% of the providers rated it as very important (Fig 1). The results also indicate that this element was reported to be the least observed or met within the VCT services. A majority of the users (71%) reported that a choice of health provider was not offered while 50% of the providers indicated that this element was rarely observed. 36% of the users experienced that there was no choice of VCT centres (Table 3).

Access to health services (in this case VCT) was mentioned as one of the sub-elements that should be covered by this element, as indicated in Table 1. The applied tool did not include questions that measure accessibility. Results from the open-ended interviews suggested that accessibility was an issue that highly influenced VCT utilization and choice of VCT centre. *"The costs of coming to the centre should be free. It is not easy to get to the VCT because it is far from my home and the transport money is expensive" (A 35 years old male urban VCT user)*. A number of informants suggested that VCT should ideally reach people in their homes or in their home environments, which could be facilitated through e.g. home-based VCT or through more mobile VCT units than are commonly offered.

### Other relevant aspects that emerged from the study

Three aspects - access to social support, continuity and follow-up, and quality of counselling and testing were not captured by the WHO tool but came up from the qualitative responses and could be essential to responsiveness in the context of VCT.

***Access to social support:*** This element was (as stated earlier) not included in our survey due to the nature of the questions included in the WHO tool. However in the present study, the open-ended responses revealed the importance of this element from a social-economic support point of view. Many informants mentioned that support groups should be linked to VCT centres to encourage more people to utilize the services. "*There should be a partnership between the VCT and the help groups to encourage more clients to come and get tested" (a 48 year female rural VCT user)*. For others, support groups were considered important in providing some provisional social economic support. *"To help us who are positive. I don't have anywhere to sleep and I have no strength" (a 48 year female urban VCT user)*.

***Continuity and Follow-up:*** Responses on follow-up care after testing and counselling were also strongly called for. Firstly, the responses indicated the need for VCT centres to attend to the users' needs and expectations in a more comprehensive manner by offering follow-up counselling both to those found positive and those found negative. Provision of drugs to those infected, provision of more condoms for prevention, family planning services and testing for other sexually transmitted diseases were also considered essential to be provided at a VCT centre. Laboratory tests were found to be a central issue "*A VCT centre should have reagents and a CD4 count machine that is working. The stopping make a portion of the clients drop out, and others suffer because in most cases people feel more free when they are taking the CD4 counts. Then they understand their viral development. That's when they get the morale to come back and start on medication" (a female urban VCT provider)*. Other informants indicated the need for all VCT centres to provide comprehensive care. *"Action should be taken on the spot. There should be no referrals to other places. Some of the places we are referred to, are very far away and we need transport to reach there" (39 year female, urban VCT provider)*.

Secondly, for the VCT centres that were integrated in a comprehensive care clinic, there was an expressed desire for reliable care by the same provider: "*those providers that work at the VCT unit should remain at the facility permanently because the movement of the providers to different department brings confusion for example related to medication of ARV follow up. Despite the records, the discussions and compromises that are not recorded are very important" (female urban VCT provider)*.

***The quality of counselling and testing:*** Various aspects related to the quality of the services emerged as central during the responses to the open-ended questions. This calls for a section that addresses quality of the services within the responsiveness tool. The number, availability and training of professional health personnel were reported by many as being insufficient. *"There should be highly trained counsellors who will counsel their clients properly so that the client leaves the place without doubts of what to do" (A 36 year male urban VCT user)*. Some expressed a lack of trust in the knowledge on HIV and AIDS provided by the providers "*The counsellors should tell the truth because some of the clients have more knowledge about HIV and AIDS than the providers" (a 27 year male urban user)*. Some of the concerns relating to the training of the counsellors that were expressed by the providers themselves confirmed the lack of sufficient knowledge. *"There is a need for refresher courses because some of the staff members were trained a long time ago. You hear them counselling clients on the corridor's but it is difficult to correct them as they are older and they feel that they know" (a female urban VCT provider)*.

## Discussion

Efforts to measure health systems responsiveness are still weak; and to our knowledge the responsiveness concept has not previously been applied to HIV-related services. In the present study, we found that all the elements that are suggested to measure responsiveness by the WHO tool deemed very important by most users and providers at VCT centres. The elements capturing respect for person's (confidentiality, dignity and autonomy) were more frequently identified as very important compared to the elements capturing client orientation (quality of basic amenities, prompt attention and choice of care providers). These results differ somewhat from a responsiveness study conducted on health systems in general in 41 countries which reported that prompt attention was the most important element, but were similar in the sense that dignity and autonomy were highly valued [32,33]. The high importance of confidentiality brought out in this study corresponds well with observations from resource-poor settings showing this element as a critical factor affecting acceptability or uptake of VCT [17,18,20-22] which is a key intervention in HIV/AIDS prevention and care [34]. Confidentiality seems to be a major factor explaining the very high acceptance rates that have been achieved when VCT has been offered at home [22,35]. Similar results were seen in a pilot study conducted in 2 districts in Kenya, which showed high acceptance of home-based HIV testing [35]. Protection of confidentiality in these settings is not only seen as an important aid to continuum of care, but crucial in reducing stigma [36].

Concern has been expressed over in the literature on how well the issue of confidentiality is handled relative to HIV testing [17,20,34,37]. The present study revealed various aspects of confidentiality that are not captured by the employed responsiveness tool. A finding that pertains to a number of elements in the tool was that the questions seem to capture the client's experiences with the health system at the time of VCT service provision inside the consultation room, but leaves out contextual aspects that may influence responsiveness substantially. Furthermore, the users expressed worries about meeting someone they knew among the clinic staff who might breach the confidentiality. This finding is in concurrence with results from various studies on factors affecting readiness and use of VCT [17,18,20], and is in particular seen as a barrier to HIV testing in places where women are prone to divorce and domestic violence if their spouses get to know they are HIV positive [38,39].

Unlike other health services, VCT services are often linked to HIV infection which is itself a stigmatized state. Stigma on the other hand affects the dignity of those infected by portraying them as persons with loose morals [40-42]. Understandably, confidentiality would be an issue of concern in the case of VCT services compared to other health services such as testing for malaria. In this case, efforts to normalise HIV testing represented by the provider initiated (opt-out) strategy are increasingly employed [43]. However, this strategy puts the process under the control of the provider when clients may not be psychologically prepared for the test. Consequently, provider-initiated testing is in greater danger of meeting with lower responsiveness than client-initiated testing done at VCT sites. The opt-out strategy has been strongly criticised for putting a low focus on counselling with the risk of undermining autonomy and reducing the focus on the preventive aspects of HIV testing [37,39,44].

Providing enough information in a language that is understandable to the users or clients is an important basis of the autonomy of the client. It was disturbing in this regard to find the informants responding to the survey questions that they were indeed informed, provided with information and given a high degree of autonomy in the counselling context, and then later to find that they understood little of the language spoken. Provider's and users of the VCT facilities expressed high expectations on provision of HIV knowledge at the facility. Providers however pointed out a need for more courses while the users indicated a lack of trust in the quality of the knowledge of HIV and AIDS among some of the providers. Information is a powerful tool in prevention and care of HIV [45], and is of critical importance in counselling processes covering both psycho-social and the preventive aspects [16]. This also challenges the responsiveness tool particularly in covering the communication process and content of information presented extensively.

Some aspects of client orientation did not appear to have been captured by the tool. Responses related to the Choice of care provider indicated that access to HIV testing has been seriously hampered by unaffordable indirect costs, such as long distances to travel to the VCT. This may in part explain the low proportion tested in high HIV prevalence countries [33]. Access to social support was also indicated as important from different perspectives. Economic support is one of the important measures of the continuum of care for HIV; if absent, it can be a barrier to better care for the people living with the virus [33].

Scaling up of HIV testing has been ongoing in many sub-Saharan countries, and in Kenya there were over 900 VCT sites in 2008 [35]. The scaling up has put priority on decentralizing VCT services as much as possible, a strategy that succeeded in making testing and preventive counselling much more easily available over a relatively short period of time. Our informants seemed concerned by the limitation of these services, revealed by the suggestions to apply the principle of continuity or follow-up of care. *"Then what, if diagnosed HIV positive or HIV negative?" *was a common question raised by users. Considering the high degrees of stigma that corresponds to fewer disclosures of HIV status [46], follow-up services and functioning referral becomes vital in this context. Comprehensive care clinics (CCC) have been established in central hospitals to offer integrated HIV services. The CCC offers a variety of services including some of what were mentioned by our informants like STIs and ART delivery [47]. From a perspective of HIV prevention, decentralised acceptable VCT services with high focus on preventive counselling should clearly be given higher priority compared to spending more on decentralising comprehensive care. However, the quality of counselling services appeared in this study to need further strengthening, and counsellors should be given a clear mandate in terms of follow up including referral. As an additional responsiveness element, continuity contributes to better quality of care [48] and is an expectation not only related to HIV infection as suggested by our respondents, but to other chronic illnesses, as described elsewhere [49]. Embracing continuity, comprehensiveness and integration within responsiveness is in line with visions of primary health-care [50].

Previous studies on responsiveness have focused mainly on the evaluation of the entire health systems within or amongst different countries [3,9,10,12,32] but our analysis concurs with other studies [11,49] in suggesting that the responsiveness concept can also be applied in specific parts of the health system. However, there are weaknesses in our study. We expected that the VCT users' views would have been more critical than the providers, as indicated in previous studies [51]. The failure to capture such a discrepancy may have been aggravated by the fact that we conducted the exit interviews at the facilities after entrusting the providers to alert the study participants. Conducting exit interviews at a VCT setting was challenging because the test results of the respondents could have emotionally influenced the responses. Some of the respondents could have given rushed responses due to the long waiting hours and the study setting could have been a challenge as opposed to home setting. In an attempt to minimize this challenge, we tried to make our respondents comfortable before embarking on the interviews and we made it clear that the test results were not important to our study.

The survey suggested a very high performance of the responsiveness elements at the VCT facilities, but mixing research methods helped us unveil issues that would not have been captured by the quantitative part of the study alone. A weakness of the qualitative part of the study was the taking of notes rather than audio-recording. Only four of these interviews were audio recorded thus, it is possible that not all the informants' statements were fully captured. We did however attempt to recode as much as possible by asking the informants to talk slowly and to repeat central messages so they could be recorded word by word. We also made observations and took photographs to visualize the settings.

## Conclusions

The findings of this study go a step further than other studies in identifying potential weaknesses in the responsiveness tool and in identifying dimensions that could be incorporated in the WHO tool. It is likely that the current findings apply more widely than to Malindi alone. The responsiveness elements proposed by WHO were all given very high ratings in the context of voluntary HIV counselling and testing. However, the study findings indicate that the tool will need substantial adjustments to capture important dimensions and perspectives. Firstly, adjustments are needed to penetrate dimensions related to the elements most valued by the respondents (confidentiality, dignity and autonomy). Secondly, there is a need to add elements that are not covered by the applied tool, such as the need to address not only dimensions inside the facility but aspects of the surrounding environment such as location of the facility potential to securing confidentiality outside the VCT room, follow-up care as well as social support. Thirdly, assessment and recognition of locally specific aspects and meanings of the elements seems of particular importance before conducting comparative studies on responsiveness of HIV testing services.

## Competing interests

The authors declare that they have no competing interests.

## Authors' contributions

MKN prepared the study proposal, coordinated and supervised data collection, conducted the interviews, analyzed the data, interpreted the findings and wrote the manuscript. AB was involved in developing the study proposal and analyzing the qualitative data, and also took an active role in interpreting the findings and revising the manuscripts. IKN contributed in developing the study proposal and took an active part in revising the manuscript. KF was involved in developing the study proposal, analyzing the quantitative part of the data and took an active part in the interpretation and revision of the manuscripts.

## Pre-publication history

The pre-publication history for this paper can be accessed here:

http://www.biomedcentral.com/1472-6963/9/243/prepub

## Supplementary Material

Additional file 1 **VCT facility located next to a general consultation room**. A photo showing how close the VCT location is to the general consultation room. The closeness of these two rooms made it almost impossible to access VCT in fear of being seen by a known provider or other users. Click here for file

Additional file 2 **A typical residential home in rural Malindi vs VCT facility**. A photo showing the kind of homes that most of the study participants live in (left) in comparison to the VCT facility (right) they were requested to evaluate. Click here for file
