# Supplementary figures and images for: A critical assessment of the WHO responsiveness tool: lessons from voluntary HIV testing and counselling services in Kenya
Source: BMC Health Serv Res. 2009 Dec 22;9:243. doi: 10.1186/1472-6963-9-243 (PMC2811110; doi:10.1186/1472-6963-9-243)

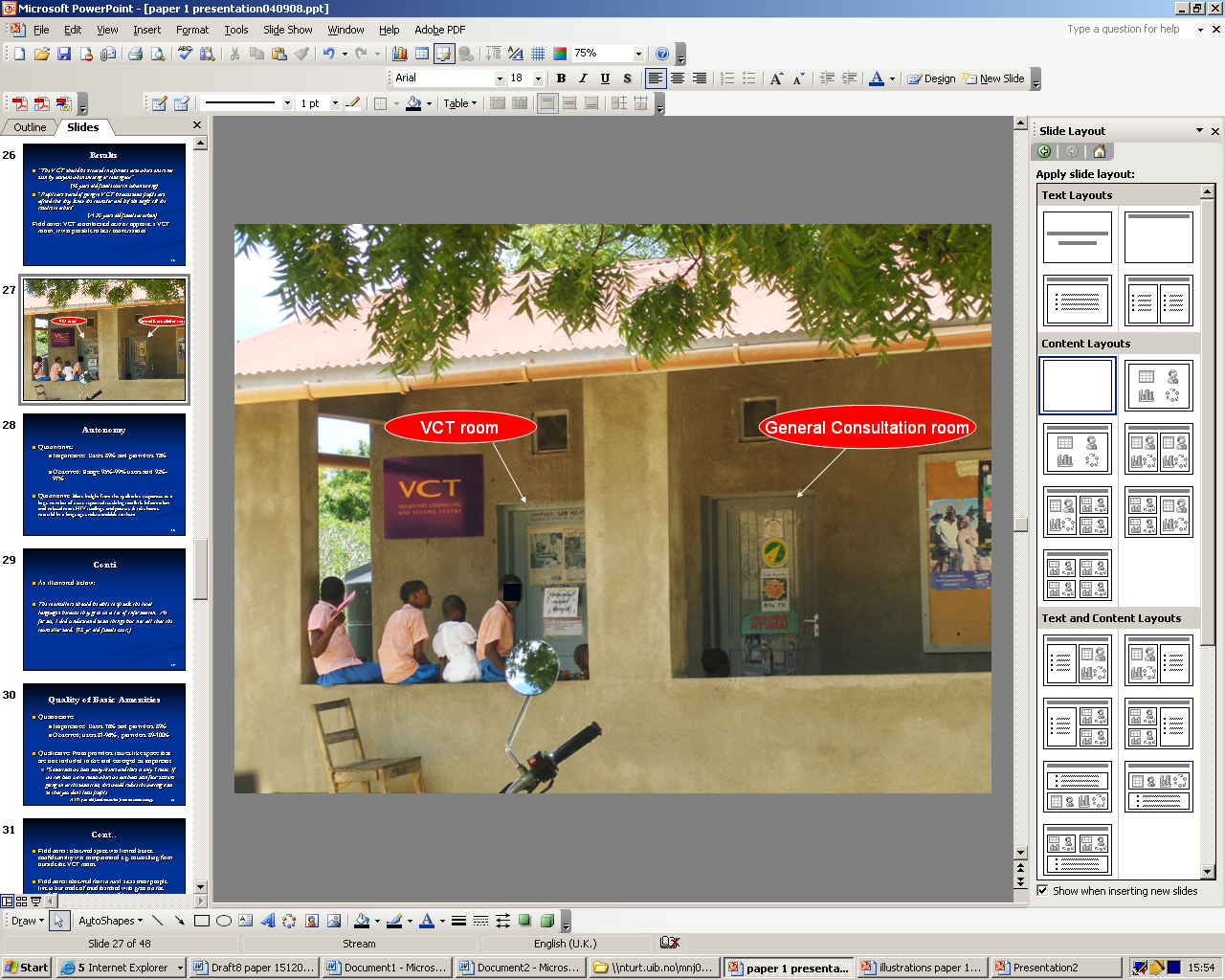

Supplement: Additional file 1 — VCT facility located next to a general consultation room. A photo showing how close the VCT location is to the general consultation room. The closeness of these two rooms made it almost impossible to access VCT in fear of being seen by a known provider or other users. [file 1472-6963-9-243-S1.DOC]

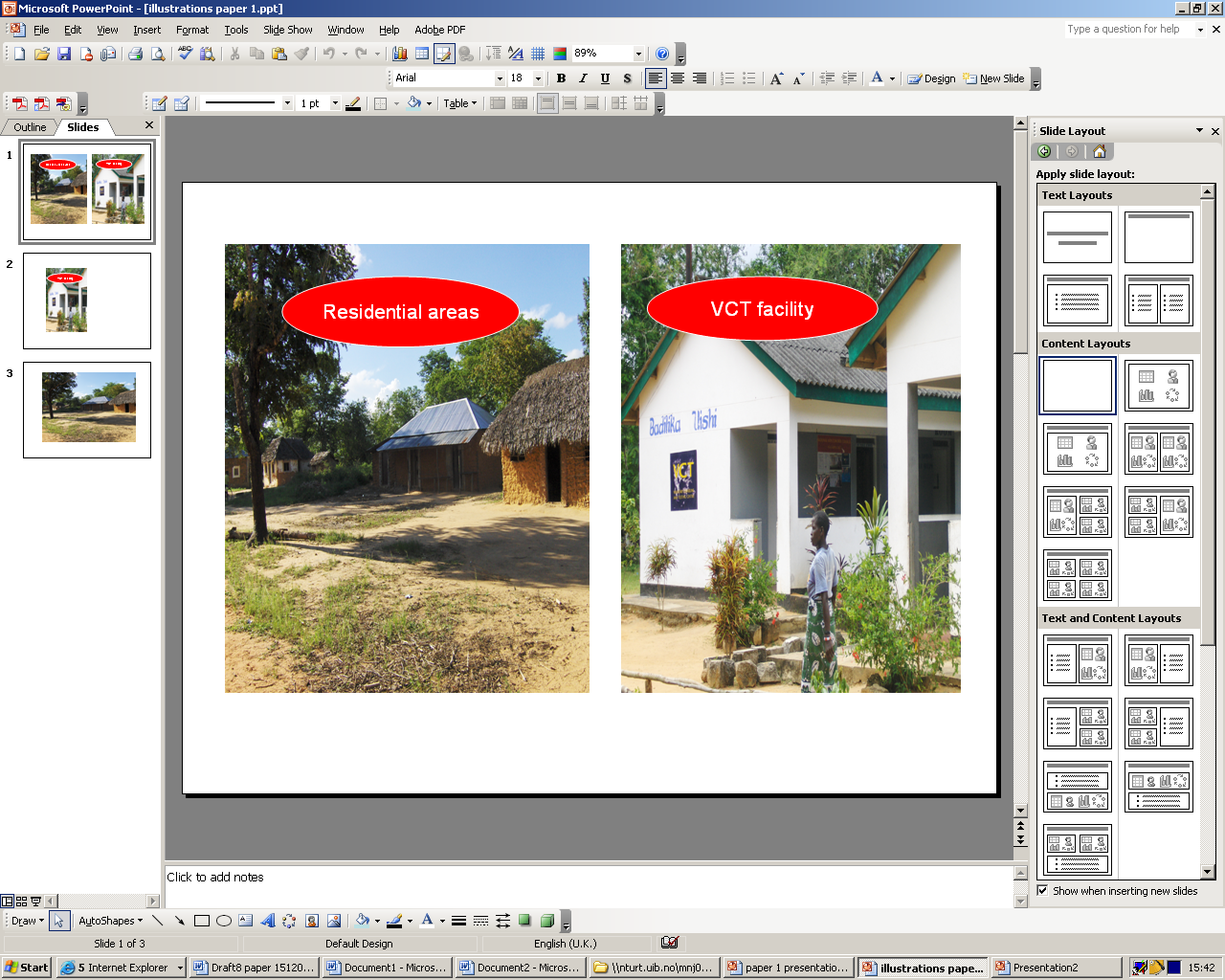

Supplement: Additional file 2 — A typical residential home in rural Malindi vs VCT facility. A photo showing the kind of homes that most of the study participants live in (left) in comparison to the VCT facility (right) they were requested to evaluate. [file 1472-6963-9-243-S2.DOC]
